# An *in vivo *investigation of the initiation and progression of subchondral cysts in a rodent model of secondary osteoarthritis

**DOI:** 10.1186/ar3727

**Published:** 2012-02-03

**Authors:** David D McErlain, Veronica Ulici, Mark Darling, Joseph S Gati, Vasek Pitelka, Frank Beier, David W Holdsworth

**Affiliations:** 1 Department of Medical Biophysics; Schulich School of Medicine and Dentistry, The University of Western Ontario, 1151 Richmond Street, London, ON, Canada. N6A 3K7; 2 Imaging Research Laboratories, Robarts Research Institute; Schulich School of Medicine and Dentistry, The University of Western Ontario, 100 Perth Drive, London, ON, Canada. N6A 5K8; 3 Department of Physiology & Pharmacology; Schulich School of Medicine and Dentistry, The University of Western Ontario, 1151 Richmond Street, London, ON, Canada. N6A 3K7; 4 Department of Pathology; Schulich School of Medicine and Dentistry, The University of Western Ontario, 1151 Richmond Street, London, ON, Canada. N6A 3K7; 5 Department of Surgery; Schulich School of Medicine and Dentistry, The University of Western Ontario, 1151 Richmond Street, London, ON, Canada. N6A 3K7

## Abstract

**Introduction:**

Subchondral bone cysts (SBC) have been identified in patients with knee osteoarthritis (OA) as a cause of greater pain, loss of cartilage and increased chance of joint replacement surgery. Few studies monitor SBC longitudinally, and clinical research using three-dimensional imaging techniques, such as magnetic resonance imaging (MRI), is limited to retrospective analyses as SBC are identified within an OA patient cohort. The purpose of this study was to use dual-modality, preclinical imaging to monitor the initiation and progression of SBC occurring within an established rodent model of knee OA.

**Methods:**

Eight rodents underwent anterior cruciate ligament transection and partial medial meniscectomy (ACLX) of the right knee. *In vivo *9.4 T MRI and micro-computed tomography (micro-CT) scans were performed consecutively prior to ACLX and 4, 8, and 12 weeks post-ACLX. Resultant images were co-registered using anatomical landmarks, which allowed for precise tracking of SBC size and composition throughout the study. The diameter of the SBC was measured, and the volumetric bone mineral density (vBMD) was calculated within the bone adjacent to SBC. At 12 weeks, the ACLX and contralateral knees were processed for histological analysis, immunohistochemistry, and Osteoarthritis Research Society International (OARSI) pathological scoring.

**Results:**

At 4 weeks post-ACLX, 75% of the rodent knees had at least 1 cyst that formed in the medial tibial plateau; by 12 weeks all ACLX knees contained SBC. Imaging data revealed that the SBC originate in the presence of a subchondral bone plate breach, with evolving composition over time. The diameter of the SBC increased significantly over time (*P *= 0.0033) and the vBMD significantly decreased at 8 weeks post-ACLX (*P *= 0.033). Histological analysis demonstrated positive staining for bone resorption and formation surrounding the SBC, which were consistently located beneath the joint surface with the greatest cartilage damage. Trabecular bone adjacent the SBC lacked viable osteocytes and, combined with bone marrow changes, indicated osteonecrosis.

**Conclusions:**

This study provides insight into the mechanisms leading to SBC formation in knee OA. The expansion of these lesions is due to stress-induced bone resorption from the incurred mechanical instability. Therefore, we suggest these lesions can be more accurately described as a form of OA-induced osteonecrosis, rather than 'subchondral cysts'.

## Introduction

Osteoarthritis (OA) is an articular joint disorder that leads to mechanical failure within the knee, causing pain and deformity. Although this degenerative joint disease is common throughout North America [1-3], there is an unmet need for pharmacological therapies that modify or reverse the structural damage and alleviate symptoms [4]. The erosion of cartilage (that is, full thickness lesions or loss of surface integrity) is commonly associated with OA [5,6]. However, changes to the underlying subchondral bone, through variations in regional volumetric bone mineral density (vBMD) or the rapid unorganized remodeling seen after injury, are likely to cause greater structural damage to joints by inhibiting local blood flow [7] and the formation of sclerosis [8,9]. Moreover, it has been shown that alterations in the biomechanical integrity of subchondral bone can modify the ability of the overlying cartilage to function normally [4,10,11]. Identifying pathological features within patients exhibiting aggressive disease progression is paramount to ensuring the proper treatment regimen is used, whether that includes physical therapy, pharmacological or surgical interventions [12].

Intra-osseous lesions, commonly known as subchondral bone cysts (SBC), 'pseudo-cysts' or 'geodes' [13,14], within OA knees have recently been associated with greater pain and disease progression. SBC were first identified by Ondrouch [15] and Landells [16] in the load-bearing regions of the femur, patella, and shoulder of arthritic patients, although the exact cause is not well known. Currently, there are two conflicting theories proposed for the origin of SBC in OA: 'synovial fluid intrusion', via a breach in the subchondral plate caused by the diminished local cartilage, leading to a rapid inflammatory response; or 'bony contusion', where stresses in the bone below the joint surface (due to trauma or thinned cartilage) exceed the functional strength of the trabecular bone, causing micro-fracture, edema, and focal bone resorption [14,15,17]. However, those earlier studies only utilized histological techniques, and were limited to analyzing tissue that was removed during a surgical procedure, such as total knee replacement [18], where the diseased or lesion-occupied area is severely degraded. Concurrently, the ability to monitor the progression of these cystic lesions *in vivo *has been limited to retrospective analyses of patients' MRI scans [18-21] which provide limited information regarding the onset of SBC formation. Given recent evidence that associates the presence of SBC, found in 25% to 47% of painful OA knees, with greater cartilage loss and increased risk of joint replacement among OA patients [19,21], it is apparent that this subchondral bone feature warrants further study.

Pre-clinical models can provide valuable insights into the cause of SBC by producing patterns of cartilage and bone degeneration that are similar to those seen in human post-traumatic or secondary OA. The surgical induction of a partial medial meniscectomy, combined with a severed anterior cruciate ligament (ACLX), in the rodent knee can emulate several aspects of secondary OA (including SBC) in a predictable and reproducible manner [22,23]. The use of preclinical imaging, specifically micro-computed tomography (micro-CT), has become routine in the past several years for longitudinal, *in vivo *monitoring of disease progression [24]. When combined with the superior soft-tissue contrast provided by high-field magnetic resonance imaging (MRI) [25,26], dual-modality imaging has the potential to characterize joint degradation comprehensively [23] and differentiate the bone, fluid, and tissue components found within SBC [16]. However, few studies have capitalized on the ability to incorporate image registration, which ensures any observations are conducted at the same three-dimensional (3D) location within each volume.

The purpose of this study was to use dual-modality, *in vivo *micro-imaging techniques to monitor the development of intra-osseous lesions occurring in an established pre-clinical model of OA. By combining our quantitative measurements with end-stage histology and immunohistochemistry, we intend to accurately determine the contents within these SBC and identify the possible existence of an open communication between the subchondral bone and joint space. Thus, these advanced analyses will provide valuable insight into the origin of OA SBC through the possible confirmation of one of the prevailing theories while simultaneously capturing the progressive remodeling of osteoarthritic bone. Furthermore, we hope to define more accurately this increasingly prevalent condition that is associated with more actively degenerative OA.

## Materials and methods

### Rodent model of secondary OA

As described previously, our preclinical model of OA involves an anterior cruciate ligament transection with partial medial meniscectomy (ACLX), performed on male Sprague-Dawley rats [22,27]. The surgery involved an incision through the medial joint compartment of the right knee, with subsequent ligament bisection and removal of the anterior horn of the medial meniscus. All animal work was conducted in accordance with the Canadian Council on Animal Care guideline, and approved by the Animal Use Committee at the University of Western Ontario. Eight rats (body weight = 416 ± 6 g, mean ± SE) were used throughout the time-course of the imaging experiments, and were permitted free activity with food and water *ad libitum *between scanning sessions. The rats were scanned with our dual-modality imaging protocol prior to ACLX, and at 4, 8, and 12 weeks post-ACLX.

### *In vivo *9.4 T MRI and micro-CT

**Figure 1:**
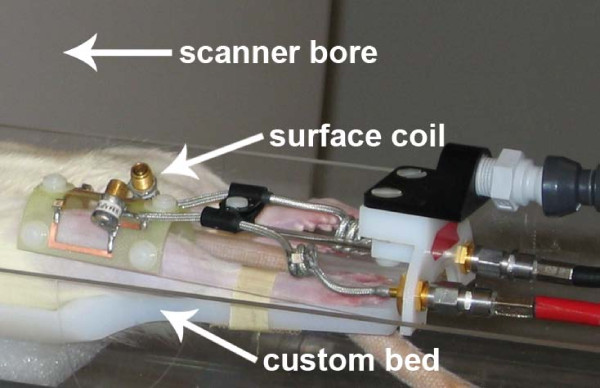
**Custom bed designed for dual-modality preclinical imaging of the knee**. Non-ferromagnetic plastic was used to position the rat with its knees extended. Upon completion of an MRI scan, the surface coil was retracted and a tissue calibration phantom (not shown) was placed along the joint line. The animal remained splinted to the bed for both MRI and micro-CT imaging sessions, which inhibited knee flexion and improved image registration. micro-CT, micro computed tomography; MRI, magnetic resonance imaging.

A custom animal bed was used for both MRI and CT scanning, where the rat's hind limbs were secured to inhibit any flexion of the knee between scans (Figure 1). This bed could be detached and re-attached to either scanner, allowing for quick transition between the MR and CT scanner facilities with little change in rat knee flexion.

All rats were anesthetized by an intramuscular injection of ketamine (100 mg/ml), xylazine (5 mg/ml) and saline prior to imaging. The dose was maintained at 0.1 ml/100 g body mass for each successive imaging session. High-field MRI images were acquired using an actively shielded, horizontal 9.4 T scanner (Varian, Palo Alto, CA, US). Slices were acquired in the sagittal plane with a 2 cm surface coil using a 3D FLASH sequence (256 × 256 × 96 matrix, TE = 3.0 ms, TR = 10 ms, FA = 5.5, 12 averages, 25.6 × 25.6 × 9.6 mm^3 ^field of view), which required approximately 32 minutes of scan time. The resultant 3D volume resolution was 0.1 × 0.1 × 0.15 mm^3^. Once MRI was complete, the surface coil was removed and a tissue-calibration phantom was placed near the joint line, prior to micro-CT imaging. This solid-state phantom contains a single insert that mimics the radiographic properties of cortical bone (SB3, Gammex RMI, Middleton, WI, US) which ensured the CT data set would be scaled in Hounsfield units and calibrated in volumetric bone mineral density (vBMD, mg/cm^3 ^of hydroxyapatite) [22,28]. Micro-CT scans were performed using a bench-top scanner (explore Locus, General Electric Health Care, London, ON, CA) with the following parameters: 210 views over 1° increments, 5 exposures averaged per view, 400 ms exposure time using 80 kV and 0.45 mA, isotropic reconstructed voxels with 0.045 mm dimensions, scan time approximately 17 minutes.

**Figure 2:**
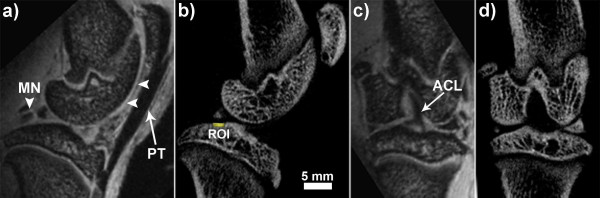
**Dual-modality imaging in an *in vivo *preclinical model of OA**. Sagittal (**a, b**) and coronal (**c, d**) plane images of the rat knee obtained from co-registered high-field MRI (a, c) and micro-CT (b, d). The MRI volume was resampled to isotropic voxel dimensions prior to co-registration, which allowed the delineation of bone and non-bone components in the meniscus (MN) and the accurate depiction of tendon (PT, patellar tendon), ligament (ACL, anterior cruciate ligament), and cartilage (horizontal arrowheads). ROI, region of interest, identifies approximate location where volumetric bone mineral density was calculated above the SBC. micro-CT, micro computed tomography; MRI, magnetic resonance imaging; OA, osteoarthritis.

The reconstructed data sets were examined in multi-planar reformatted view and all measurements were performed on a workstation using MicroView (v.2.11, General Electric Healthcare, London, ON, CA) software. Due to unequal voxel sizes between the MRI and micro-CT data sets, the MRI data was resampled, using in-house software, to an isotropic voxel dimension of 0.045 mm which allowed for anatomical landmark-based, rigid-body registration [25] and the creation of a dual-modality image of the knee (Figure 2). The presence of cysts was detected in all three anatomical planes, however, the coronal plane was chosen when the maximum diameter of the lesion was determined, which was defined as the maximum extent of focal loss of bone in the medial-lateral direction within the affected joint compartment. Thus, for each rat knee volume per time point, the user identified the lesions as focal areas of bone loss that were spherical or ellipsoidal in shape and appeared adjacent to the joint surface of either the femur or tibia. Upon verification that a SBC had been identified within the dual-modality images, vBMD measurements of the subchondral bone, in the region between the SBC and the articular surface, were acquired using the region of interest (ROI) tool within the micro-CT volume. Cylindrical ROIs, with a diameter of 0.68 mm and a height of 0.23 mm, were placed in the superficial regions of bone and values for vBMD were calculated within each rodent knee over time. Therefore, an accurate depiction of the changes to the bone, which occurred *in vivo *as OA progressed and the SBC modified its size, was obtained. In addition, the maximal diameter of the lesion was recorded from the CT data, while the corresponding MRI section was utilized in an attempt to identify the heterogeneous tissue types, such as a fibrous or fluid-filled cavity, that appeared within the lesion space. Finally, the diameter of the SBC, occurring in each OA rat knee, was compared between time points to allow for longitudinal analysis of lesion size and any possible changes in SBC composition.

### Histological end-stage analysis

At 12 weeks post-ACLX, the rats were euthanized by an intraperitoneal injection of pentobarbital (110 mg/kg body weight). Both hind limbs were disarticulated and decalcified in 15% EDTA/glycerol (pH = 7.3) for up to 5 weeks. The knee joints were subsequently embedded in paraffin wax and the medial joint compartment was serially sectioned in the sagittal plane. Sections were selected based on their proximity to the SBC that were identified and measured from the medial bony joint margin in the micro-CT images. A minimum of three sections, each 5 μm thick, were stained with Safranin-O and fast green, and the Osteoarthritis Research Society International (OARSI) scoring method was used to grade and stage OA degradation in the medial compartment of both the ACLX and the contralateral knee [29]. The OA score was calculated by multiplying the grade and stage values for each slide (a minimum score of 0 represents no OA degradation and a score of 24 represents the maximum degree of OA) and the total score was achieved from the average of the mean score of all slides (≥ 3) for each joint surface.

Chemical reagents were purchased from Sigma-Aldrich (Oakville, ON, CA), unless stated otherwise. Antibodies were purchased from Abcam (alkaline phosphatase - ALPL # ab95462; Cathepsin K # ab37259, Cambridge, MA, US), and Santa Cruz (HRP conjugated goat anti-mouse # sc-2005, HRP conjugated goat anti-rabbit # sc-2004, Santa Cruz, CA, US). DAB substrate-chromogen was purchased from Dako (K3468, Burlington, ON, CA). Knee joint sections, each 5 μm thick, were dewaxed, re-hydrated (xylene, 100% ethanol, 95%, 70% and H_2_O), and stained with hematoxylin and eosin, Safranin O and fast green and Picrosirius red, using standard protocols [30,31].

Immunohistochemistry protocols were performed as previously described [32] with some modifications. Rehydrated sections were incubated in 3% hydrogen peroxide for 15 minutes at room temperature, followed by heat antigen retrieval; the slides were incubated with preheated (2 minutes at 100°C) 10 mM sodium citrate solution (pH = 6.0) for 30 minutes at 97°C (for ALPL antibody). In the case of Cathepsin K antibody, incubation with 0.1% TritonX-100 for 10 minutes was used for antigen retrieval. Subsequently, they were blocked with 5% goat serum and incubated with primary antibodies against ALPL and cathepsin K over night at 4°C, followed by HRP conjugated- secondary antibody for 1 hour at 4°C. After washing, the sections were incubated for 2 to 5 minutes with diaminobenzidine (DAB) substrate solution, washed and mounted. Counter-staining was performed with methyl green for 5 minutes, which was followed by dehydration and mounting of the sections. Images were taken with a Retiga EX camera (QImaging, Surrey, BC, CA), connected to a DMRA2 microscope (Leica microsystems, Wetzlar, Hesse, DE). Primary image analyses were performed using Openlab 4.0.4 and Adobe Photoshop software.

### Statistical Analysis

Quantitative values for SBC diameter were compared using a one-way analysis of variance (ANOVA) with a Tukey's post-hoc test as there were unequal n-values per time point due to the formation of additional SBC detected after four weeks post-ACLX. The vBMD data above the SBC that appeared throughout the whole course of the study were compared using a repeated measures ANOVA. Differences for each parameter, between all time points, were considered significant for *P *< 0.05. The OARSI grade, between the ACLX and contralateral limb of the same animal, was compared using a Wilcoxon signed rank test.

## Results

### Incidence and Appearance of SBC

**Figure 3:**
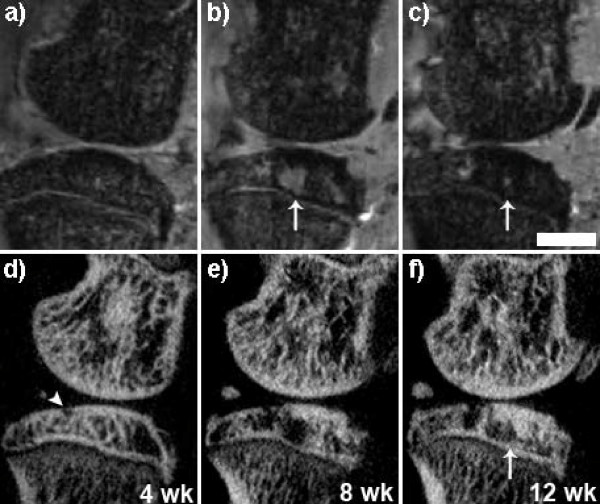
**Longitudinal monitoring of subchondral bone cysts (SBC) developing in the medial tibial plateau**. Co-registered sagittal 9.4 Tesla MRI (**a-c**) and micro-CT (**d-f**) sections reveal the onset of SBC formation in conjunction with a breach of the subchondral bone plate (d - arrowhead), which never fully repairs over time. All SBC expanded over time, 4 weeks (wk) to 12 wk post surgery, and appeared to transition from a vacuous, fluid filled cavity (b - arrow) to a more fibrous filled region containing immature or disorganized osteoid (c, f - arrows). Scale bar = 2 mm. micro-CT, micro computed tomography; MRI, magnetic resonance imaging.

**Figure 4:**
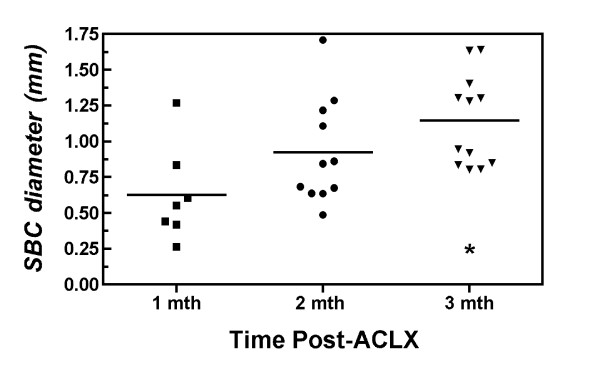
**Mean maximal diameter of subchondral bone cysts (SBC) in the medial tibial plateau for all rodents post surgery**. Points represent the SBC measured in the coronal plane using micro-CT at each time point. Repeated measures ANOVA found the diameter increased significantly at 3 months post-ACLX (anterior cruciate ligament with partial medial menisectomy) versus the 1-month time point (*P < 0.05). ANOVA, analysis of variance; micro-CT, micro computed tomography.

The *in vivo*, dual-modality imaging protocol was completed in less than one hour using a single dose of anesthetic. The reconstructed and registered data sets provided excellent visualization of the major structural components in the rat knee including the subchondral bone, articular cartilage, and meniscus, which contains both bone and non-bone components (Figure 2). The presence of SBC was found in all animals throughout the time-course of the study (12 weeks) and primarily occurred in the medial tibial plateau (MTP). Some SBC were identified within the medial femoral compartment, however these occurred less frequently, and therefore we will only discuss SBC within the MTP. Focal areas of bone resorption, with subsequent loss of bone mineral density (Table 1), were identified initially in the micro-CT volume among 6 of 8 rat knees (75%) at 4 weeks post-ACLX, and all knees contained at least one cyst by the endpoint. In addition, a second cyst appeared in 63% (that is 5 out of 8) of ACLX rats by the final time point (12 weeks). No intra-osseous lesions were found in the contra-lateral (non-operated) limb of ACLX animals throughout the study. The SBC were primarily pyriform or spherical shaped and were located underneath a breach of the subchondral bone plate (Figure 3) that was defined as an open communication between the joint space and trabecular bone through the subchondral plate, and was more easily distinguished in the micro-CT volume due to its higher spatial resolution (Figure 3a). The MRI revealed a heterogeneous signal within the SBC that was higher at earlier time points, though not as high as cartilage, and gradually degraded over time revealing a transition from a more fluid-filled to fibrous-filled cavity (Figure 3b, c). The mean diameter of the SBC significantly increased over time (*P *= 0.013), and Tukey's post-test revealed a significantly larger diameter at 12 weeks post-ACLX versus 4 weeks (Figure 4, *P *< 0.05). The micro-CT derived SBC diameters were usually higher than the MRI measurements (Table 1), though overall the values agreed within 10%.

**Table 1:** Descriptive statistics for all operated (ACLX) knees containing subchondral bone cysts (SBC) (Number = 8)

Time post-ACLX	CT diameter (mm)	MRI diameter (mm)	vBMD (mg/cc)	OARSI grade
**Presurgical**			770 ± 19	
**4 weeks**	0.626 ± 0.126	0.656 ± 0.0781	680 ± 52	
**8 weeks**	0.922 ± 0.111	0.827 ± 0.105	610 ± 37*	
**12 weeks**	1.14 ± 0.0923*	1.07 ± 0.0809*	690 ± 57	18 ± 0.82 2.9 ± 0.58

Values are mean ± standard error from the measured SBC diameter and superficial volumetric bone mineral density (vBMD) occurring over time. OA grade (OARSI), acquired from the histological analysis, is provided for the final time point only and presented versus the contralateral limb score underneath. *vBMD = *P *< 0.05 versus presurgical, *diameter = *P *< 0.05 versus 4 weeks. ACLX, anterior cruciate ligament transection with partial medial meniscectomy; CT, computed tomography; MRI, magnetic resonance imaging; OARSI, Osteoarthritis Research Society International.

### Quantitative and Histological Analysis

**Figure 5:**
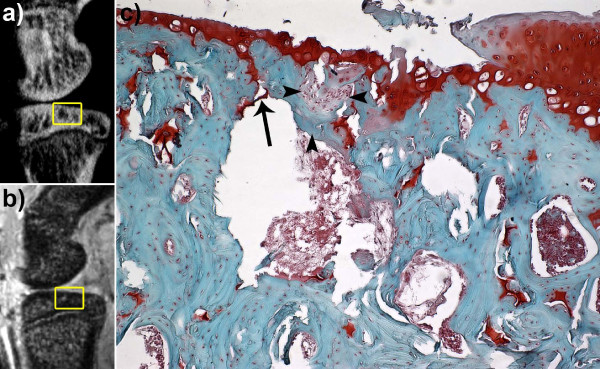
**Identification of the subchondral bone plate breach found in subchondral bone cysts (SBC) in the OA rat knee**. Sagittal micro-CT (**a**) and high-field MRI (**b**) sections were used to observe and monitor SBC *in vivo*. End stage histological analysis confirmed separation in the subchondral bone plate (**c **- arrow, 10 × magnification of Safranin-O with fast-green counter stain). Note, the fibrocartilage repair tissue adjacent the SBC (horizontal arrowheads) and breach to the subchondral plate. Remaining bone tissue between the repair tissue and the SBC lacks the presence of osteocytes (vertical arrowhead) that indicates focal necrosis. Note the 'pear' shape of the intra-osseous lesion, consistent with SBC in humans. micro-CT, micro computed tomography; MRI, magnetic resonance imaging; OA, osteoarthritis.

**Figure 6:**
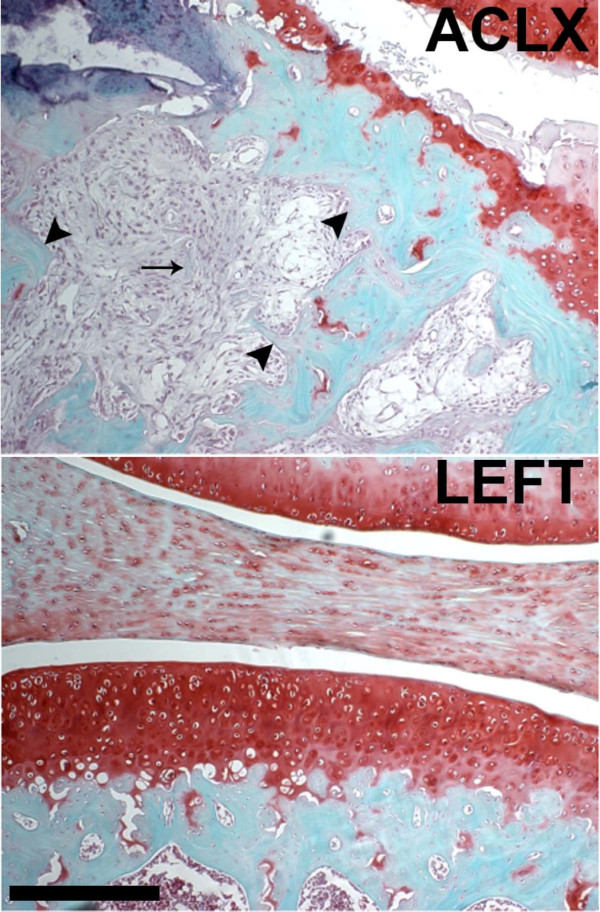
**Safranin-O stained histological section of the operated (ACLX) and un-operated knee (LEFT) 3 months post-surgery**. Note, the remaining fragment of trabecular bone (left, arrow) within the cyst region and the lack of viable osteocytes within the surrounding bone (arrowheads), which indicates focal osteonecrosis. Scale bar = 250 μm. ACLX, anterior cruciate ligament transection with partial medial meniscectomy.

**Figure 7:**
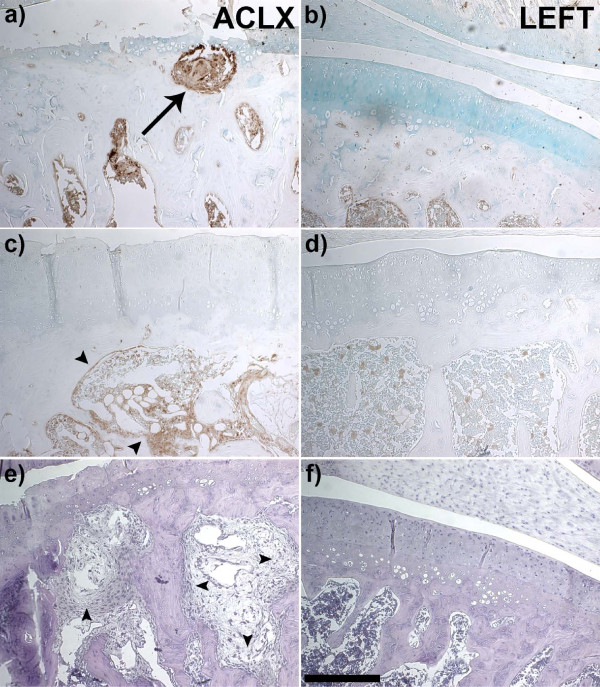
**Histological characterization of cyst development in a rodent model of Osteoarthritis**. Immunohistochemistry revealed an active bone remodeling response within the subchondral bone cysts (SBC), as the anterior cruciate ligament transection with partial medical menisectomy (ACLX - **a, c, e**) knees showed greater staining for markers of both resorption (Cathepsin K, a-arrow), and bone formation (Alkaline phosphatase, c-arrowheads) against the un-operated knees of the same animals (LEFT, **b, d, f**). Hematoxylin and eosin staining (e, f) showed the SBC (3 months post-ACLX) as a fibrous cavity, with fibrotic bone marrow in the ACLX knee (e-arrowheads). Scale bar = 250 μm.

vBMD was calculated within the superficial subchondral bone with cylindrical ROIs that had a total volume of approximately 0.070 mm^3^. Repeated measure ANOVA revealed that the mean vBMD per time point was significantly different (Table 1, *P *= 0.027) immediately above the SBC. Tukey's post-hoc test indicated that the decreased vBMD 8 weeks post-ACLX was significantly lower than presurgical values (*P *< 0.05). At 12 weeks post-ACLX, there was a 13% increase in peri-cystic vBMD versus 8 weeks post-ACLX; although it was not considered statistically significant (Table 1), this increased vBMD indicated ongoing bone remodelling. The OARSI score for the ACLX knees was significantly higher (*P *= 0.008) than the contralateral knee of the same animal (Table 1). In addition, the SBC were located at the site within the histological section that received the highest (that is, the most severe OA) grade, often accompanied by superficial or deep lesions and severe proteoglycan loss within the cartilage itself (Figure 5, 6, Additional file 1). Although difficult to identify in every section, a connection or breach of the subchondral plate was confirmed for all SBC located near the joint space (Figure 5c). However, these gaps were not an open communication with the joint space that could allow fluid exchange, but were rather filled with a fibrous or cartilaginous tissue, which seemed reparative. Areas of bone surrounding the SBC appeared necrotic and lacked any normal marrow components when compared with the contralateral limb (Figure 6, Additional files 1, 2). Moreover, there was no epithelial lining observed along the SBC margins. Immunohistochemistry revealed that even at an advanced stage of OA, evidenced by the near complete loss of cartilage, the area surrounding the SBC was still undergoing bone remodeling, with increased markers for both osteoblasts (ALPL) and osteoclasts (cathepsin K) (Figure 7).

## Discussion

To the best of our knowledge, this is the first study to indentify and monitor SBC as they occur in a preclinical model of OA. The induction of OA through surgical destabilization in the rat knee produced SBC in a consistent manner within the medial tibial plateau, which could be measured with medical imaging techniques similar to those used in humans. High-field MRI aided in the depiction of SBC having both fluid and fibrous tissue components as they increased in size and number and the rapid acquisition time enabled us to obtain an *in vivo*, dual-modality images of the rat knee in under an hour. Using histological analyses and a grading system of OA severity designed for humans [33], we found that SBC occur at the site of greatest disease severity, which agrees with previous findings in humans [21]. This study further validates the rat ACLX model of secondary OA with SBC development.

The presence of a breached subchondral plate was identified by Landells and proposed to be the source of SBC formation [16]. As synovial fluid entered the cavity, it was thought to increase the intra-osseous pressure and lead to subsequent cyst expansion. However, this is unlikely for the following reasons: the defect in the subchondral plate is small relative to the size of the SBC (Figure 5c); the course, if it existed, from joint space to SBC is tortuous or oblique to the surface, limiting the ability of easy fluid exchange (Figure 5c); the defect is filled with fibro-cartilage (Figure 5c, 7a), which is less stiff than bone, but may not allow fluid flow in the same manner as hyaline cartilage behaves as a viscoelastic solid under weight-bearing. In addition, as the SBC were still expanding by the final time point, there was evidence of immature bone deposition in some knees that would further inhibit fluid flow (Figure 3f). Thus, Ondrouch proposed that due to a thinned cartilage layer, normal weight-bearing forces within the adjacent bone are increased beyond its physiological limits, causing focal damage, subsequent resorption and SBC formation [15]. Recent mechanical simulation studies have confirmed higher intra-osseous stress values beneath the joint surface when the amount of overlying cartilage is diminished [17], which could also lead to SBC formation in the opposing bone in the joint. Our results indicate that the SBC are developing and expanding due to the mechanical instability imposed by the ACLX. Increased shear stress on the surface of the cartilage, due to the loss of the ligament, combined with a medial compartment-weight distribution bias, due to the meniscectomy, caused cartilage reduction or fibrillation and increased intra-osseous stress simultaneously. Therefore, the defect in the subchondral bone is likely a micro-fracture in response to the overloaded region in the joint. Repeated loading of this area during gait prevents sufficient healing and the space remains occupied with fibrous tissue (Figures 6, 7). Consequently, the bone below undergoes further damage leading to focal osteonecrosis, which is defined as a lack of osteocytes in the damaged area followed by the formation of fibrotic, avascular marrow [34,35].

The expansion of these SBC in OA joints occurs due to stress-induced bone resorption of necrotic tissue. In the hip, this can continue until the majority of the epiphyseal trabecular bone in the femoral head or acetabulum is resorbed, causing great pain and deformity [14,16,17]. The SBC in knee OA tend to be smaller and contain an outer rim of sclerotic bone that makes them distinguishable even when imaged with planar radiographs [13,36]. This may indicate a form of self-limitation within the bone, where the SBC become more spherical and are surrounded by a necrotic shell to help dissipate the increased loading. This may explain how some SBC can resolve or reduce in size over time [21] if the fibro-cartilage tissue within the cavity is provided the opportunity to mineralize. Sclerotic bone formation is emulated in our rat model as vBMD increased by 13% (at 12 weeks post-ACLX) on the superficial surface of the SBC after a significant decrease in vBMD at 8 weeks post-ACLX versus presurgical values. Furthermore, simulations of SBC within the human knee using finite-element analysis indicated that intra-osseous stress concentrations were higher near the joint surface [28], which could explain the sclerotic bone formation seen in the proximal tibia (Figure 2f).

One of the features lacking in our SBC models was the detectable presence of bone marrow edema, which is commonly associated with human SBC development [18-20]. A diffuse, and high-intensity fluid-like, signal upon MRI examination of the trabecular bone is the proposed evidence of edema in the joint compartment, typically as a result of injury and the subsequent initiation of the bone remodeling process [20]. Edema has been found to precede SBC formation in most cases [20,21], but its presence does not guarantee trabecular remodeling when compared with histological examination [37]. The inability to detect bone edema may have resulted from the MRI sequence used, as it did not incorporate fat suppression techniques that enhance edema visualization against the fatty bone marrow [38]. However, we were concerned about masking any marrow components that may be included within the SBC and chose not to use fat suppression in our MRI protocol. In addition, allowing a shorter duration between imaging sessions (that is, two weeks) may improve our detection of edema in the early stages of OA progression and SBC formation. With the advancement of custom designed rat knee coils [39] that incorporate longer scan times for increased signal-to-noise ratio, it will be feasible to further quantify the tissue composition of SBC over time, without reliance on tissue-destructive, end-stage histological analysis. This is further evident in the recent work using the rat model of secondary OA, combined with dual-modality imaging that demonstrated the utility of longitudinal imaging when evaluating the effect of anti-inflammatory drugs on disease progression [23].

## Conclusions

In summary, the rat ACLX model of post-traumatic knee OA provides valuable insights into the cause and composition of a degenerative feature within the subchondral bone that is associated with greater pain and disease severity. Subchondral cysts, as they are commonly referred to, appeared in a predictable manner within the overloaded joint compartment of the medial tibial plateau. These SBC do not contain any evidence of an epithelial lining and therefore are often referred to as 'pseudo-cysts' or geodes, indicating the formation of a lesion in response to stress [13,14]. Our evidence suggests that these spherical or pyriformal intra-osseous lesions contain fibrous tissue, which may initially contain fluid and can ossify in later stages, surrounded by regions that lack osteocytes and can possibly form a sclerotic margin in response to repeated, pathological loads. Therefore, we feel further studies are warranted to elucidate the unique pathogenesis of SBC that focus on the presence of focal, OA-induced osteonecrosis. A breach of the subchondral bone plate was found overlying all SBC and we believe the expansion of these SBC is more likely due to micro-fracture and stress-induced bone resorption than the influx of fluid through the breach. It remains unclear whether SBC first initiate due to a loss of cartilage integrity or as a result of a subchondral bone failure, but this debate is common in OA research and future studies should strive to characterize the response of both tissues to pathological stress conditions.

In addition, it should be noted that SBC tended to appear in combination with greater cartilage severity scores. Finally, *in vivo *dual-modality imaging proved an invaluable method for quantifying the efficacy of therapeutic interventions developed to treat SBC in humans [40] or animals [23].

## Abbreviations

ACLX: anterior cruciate ligament transection with partial medial meniscectomy; BV/TV: bone volume fraction; FION: focal: ischemic osteoarthritis-induced osteonecrosis; micro-CT: micro-computed tomography; MRI: magnetic resonance imaging MTP: medial tibial plateau; OA: osteoarthritis; OARSI: Osteoarthritis Research Society International; ROI: region of interest; SBC: subchondral bone cysts; vBMD: volumetric bone mineral density; 3D: three-dimensional.

## Competing interests

The authors declare that they have no competing interests.

## Authors' contributions

DDM conceived of the study, developed the imaging protocols, performed all imaging experiments, statistical analyses, and drafted the manuscript. VU performed histological and imaging analyses, and drafted portions of the manuscript. MD performed histological analyses. VP was involved in the development of the animal model, assisted in imaging experiments and histological analysis. FB was involved in coordination of the study. DWH was involved in the conception and coordination of the study and design of the imaging protocols. ll authors read, edited, and approved the final manuscript.

## Supplementary Material

Additional file 1 **Coronal section of the OA rat knee stained with Picrosirius Red (5× magnification)**. Subchondral cysts (SBC) within the medial tibial plateau stained positively for the presence of collagen within the lesion itself (black arrows). This confirms that the composition of the focal areas of bone loss, observed in co-registered micro-CT and MRI images, have a different composition than the normal marrow (yellow arrow). SBC appeared adjacent to the joint surface and were characteristically found in the region of the knee with the most severe cartilage degradation. Click here for file

Additional file 2 **Hematoxylin-eosin stain of the operated (ACLX) and un-operated (LEFT) knee of the same rat, 12 weeks post-surgery (10× magnification)**. Note the disorganization of the trabecular bone seen in the ACLX (arrow) versus the LEFT knee that occurred immediately adjacent the subchondral bone cyst (SBC). Click here for file
