# Supplementary figures and images for: An in vivo investigation of the initiation and progression of subchondral cysts in a rodent model of secondary osteoarthritis
Source: Arthritis Res Ther. 2012 Feb 3;14(1):R26. doi: 10.1186/ar3727 (PMC3392819; doi:10.1186/ar3727)

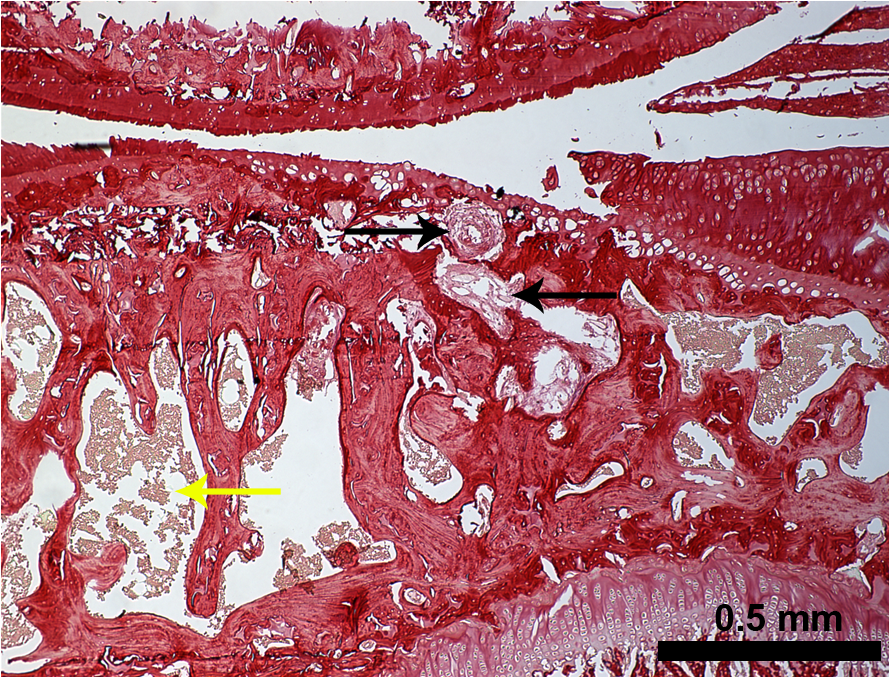

Supplement: Additional file 1 — Coronal section of the OA rat knee stained with Picrosirius Red (5× magnification). Subchondral cysts (SBC) within the medial tibial plateau stained positively for the presence of collagen within the lesion itself (black arrows). This confirms that the composition of the focal areas of bone loss, observed in co-registered micro-CT and MRI images, have a different composition than the normal marrow (yellow arrow). SBC appeared adjacent to the joint surface and were characteristically found in the region of the knee with the most severe cartilage degradation. [file ar3727-S1.TIFF]

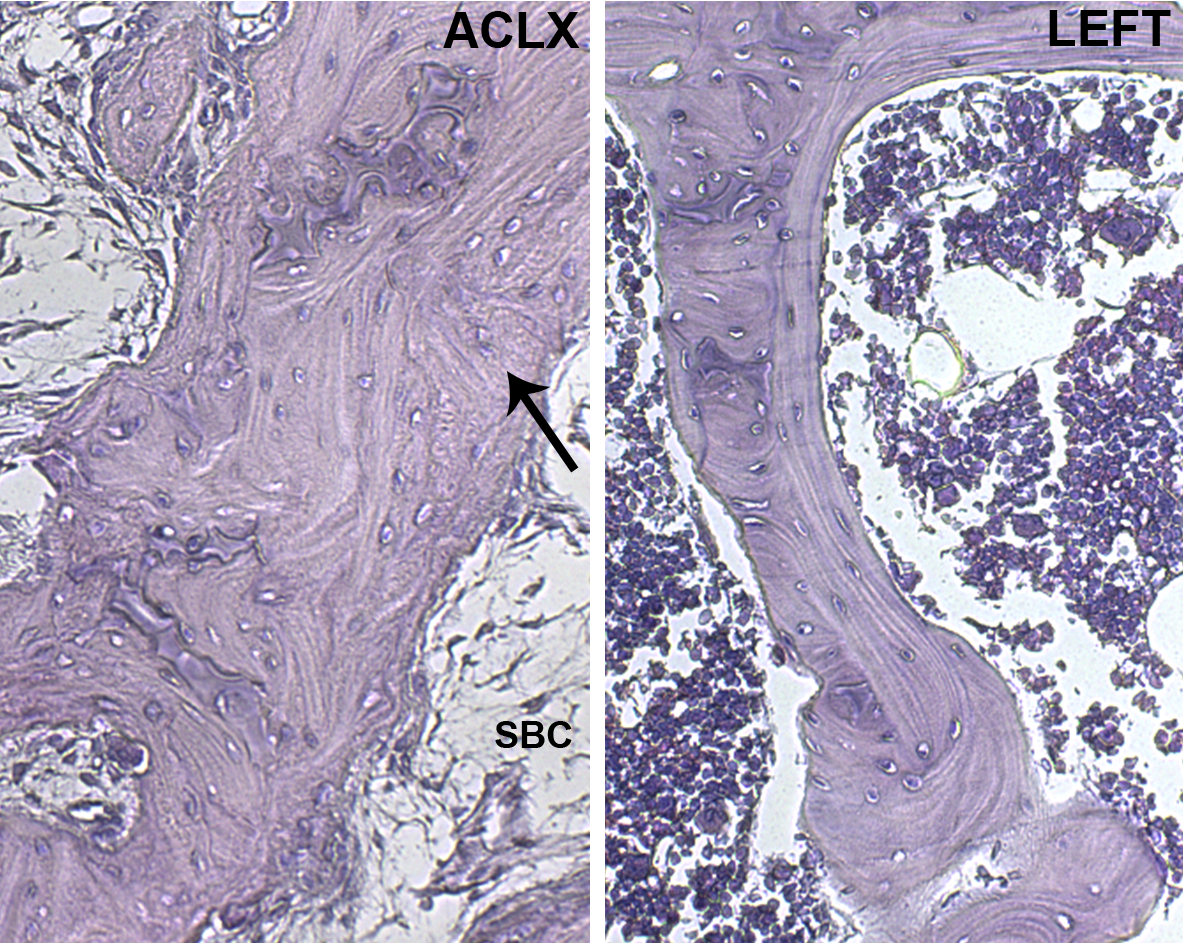

Supplement: Additional file 2 — Hematoxylin-eosin stain of the operated (ACLX) and un-operated (LEFT) knee of the same rat, 12 weeks post-surgery (10× magnification). Note the disorganization of the trabecular bone seen in the ACLX (arrow) versus the LEFT knee that occurred immediately adjacent the subchondral bone cyst (SBC). [file ar3727-S2.TIFF]
